# Clinical relevance of alerts from a decision support system, PHARAO, for drug safety assessment in the older adults

**DOI:** 10.1186/s12877-019-1179-y

**Published:** 2019-06-11

**Authors:** Khedidja Hedna, Marine L. Andersson, Hanna Gyllensten, Staffan Hägg, Ylva Böttiger

**Affiliations:** 1 Department of Medical and Health Sciences, Division of Drug Research, Linköping University, Linköping, Sweden; 2 Centre of Ageing and Health (AgeCap), Department of Psychiatry and Neurochemistry, Institute of Neuroscience and Physiology, Sahlgrenska Academy, University of Gothenburg, Gothenburg, Sweden; 3 Division of Clinical Pharmacology, Department of Laboratory Medicine, Karolinska Institutet, Karolinska University Hospital, Stockholm, Sweden; 4 Division of Insurance Medicine, Department of Clinical Neuroscience, Karolinska Institutet, Stockholm, Sweden; 5 Institute of Health and Care Sciences, Sahlgrenska Academy, University of Gothenburg, Gothenburg, Sweden; 6 Centre for Person-centred Care (GPCC), University of Gothenburg, Gothenburg, Sweden; 7 Futurum, Region Jönköping County, Jönköping and Department of Medical and Health Sciences, Division of Drug Research, Linköping University, Linköping, Sweden

**Keywords:** Adverse drug reaction, Clinical decision support system, Older adults, Drug interactions, Validity

## Abstract

**Background:**

PHARAO is a decision support system developed to evaluate the risk for a set of either common or serious side-effects resulting from a combination of pharmacodynamic effects from a patient’s medications. The objective of this study was to investigate the validity of the risk scores for the common side-effects generated by PHARAO in older patients.

**Methods:**

Side-effects included were sedation, constipation, orthostatic symptoms, anticholinergic and serotonergic effects. The alerts generated by PHARAO were tested in 745 persons ≥65 years old. Dispensed prescriptions retrieved from the Swedish prescribed drug register were used to generate the pharmacological risk scores of patients’ medications. Symptoms possibly related to side-effects were extracted from medical records data.

**Results:**

The PHARAO system generated 776 alerts, most often for the risk of anticholinergic symptoms. The total specificity estimates of the PHARAO system were 0.95, 0.89 and 0.78 for high, intermediate and low risk alerts, respectively. The corresponding sensitivity estimates were between 0.12 and 0.37. The negative predictive value was 0.90 and the positive predictive value ranged between 0.20–0.25.

**Conclusions:**

The PHARAO system had a high specificity and negative predictive value to detect symptoms possibly associated with the of patients’ medications, while the sensitivity and positive predictive value were low. The PHARAO system has the potential to minimise the risk of over-alerts in combination with a drug-drug interaction alert system, but should be used in connection with a medical evaluation of the patient.

**Electronic supplementary material:**

The online version of this article (10.1186/s12877-019-1179-y) contains supplementary material, which is available to authorized users.

## Background

Suboptimal drug prescribing is recognised as a major cause of adverse drug events among older patients [1, 2]. Prescribing is particularly challenging in the older population as they often suffer from multiple chronic diseases, use several medications concomitantly, and have increased sensitivity to drug effects [3, 4]. Moreover, the number of drugs used by older adults has increased over time [5], which increases the risk of adverse drug events due to additive pharmacological effects and drug-drug interactions (DDIs) [6, 7].

Numerous computerised decision support systems (CDSSs) for drug prescribing have been developed in order to promote rational drug use and improve patient safety [8, 9]. An optimal CDSS should have a reasonable balance of warnings with low risk of both over-alerts and too few alerts [10, 11]. Over-alerts may lead to alert fatigue and prescribers’ subsequent overriding of pertinent warnings, while too few alerts increase the risk of overlooking possible harms and may also lower the prescriber’s perception of reliability and usefulness of the system [12, 13]. The trade-off between sensitivity and specificity of such an alert system is thus highly relevant for its use in clinical practice.

We have previously developed a DDI database, SFINX (Swedish Finnish Interaction X-referencing), to be integrated in CDSSs coupled to electronic prescribing [14]. This system contains information on more than 20,000 pairs of drugs that may potentially interact adversely, and is now in use in almost all electronic medical record systems in both Sweden and Finland, as well as in several other countries. In order to decrease the risk of alert fatigue, we have had a restrictive approach to what drug pairs should be included into the SFINX database. We have been especially restrictive in entering pharmacodynamic interactions that are evident from the pharmacological mechanism of action of the substances, as these interactions would quickly multiply the content of the database, increasing the risk for over-alerting.

Furthermore, all existing DDI databases are based on drug pairs, whereas patients may have several drugs that interact concomitantly by way of additive or synergistic drug effects, in addition to the risk of pharmacokinetic interactions. Therefore, the PHARAO (Pharmacological Risk Assessment Online) system was developed as a complement to the SFINX database in relation to potential pharmacodynamic interactions, taking into account the combinations of more than two drugs, and yet minimising the risk of alert fatigue. By adding information on pre-specified pharmacodynamic properties of substances that may give additive or synergistic effects, a risk profile for the individual patient’s drug list can be created, without compromising the specificity of the drug pair alerts [15]. The risk profile should preferably be generated on demand by the user, such as during the medication review, and not appear as automated alerts. The PHARAO system is presently available in Finland and other countries through the distribution by medBase Ltd. and in the capital region of Sweden (as *Janusmed Riskprofil*) by the Stockholm County Council.

However, there is a lack of evidence on the relevance of CDSS signals to detect actual adverse dug events or other drug-related problems experienced by patients. There is therefore a need to investigate the association between the CDSS generated information and actual symptoms experienced by patients. The objective of this study was to investigate the validity of the pharmacological risk profile generated by PHARAO (presently available as Janusmed Risk Profil in Sweden) in an old adult population, based on patients´ experienced symptoms as identified in medical records. The specific aim was to investigate how well the risk warnings correlated with symptoms that may be associated with the pharmacological properties of prescribed drugs.

## Method

### Setting and population

The study used data from the DRUMS project (Drug-related Morbidity in Sweden). The study included 745 persons aged 65 years and older identified from a previous population-based cohort of 5025 adult residents (≥18 years on 31 December 2007) in Östergötland Region, Sweden. The source population had healthcare encounters in 110 specialised outpatient clinics, 51 primary care units, and 29 departments of inpatient care in three hospitals [16]. The old adults who had at least one healthcare encounter and refilled at least one prescribed medication over a 3-month period in 2008 were included in the study.

### Data sources

Data from several sources were linked using the personal identity number [17]. Data on prescribed medications refilled during the 3-month period were extracted from the Swedish Prescribed Drug Register. This register covers all prescribed drugs dispensed to outpatients in pharmacies, including prescription drugs used in residential care and nursing homes [18]. Data on healthcare encounters were retrieved from the regional patient register, the Care Data Warehouse in Östergötland. This register includes all public and most private healthcare encounters conducted in the region [19]. Medical records from all healthcare providers identified through the register were reviewed for unwanted symptoms of any kind.

### Evaluation of the pharmacological risk scores of refilled prescribed medications

The calculation of the pharmacological risk scores of patients’ medications was based on the PHARAO decision support system. This system consists of a database with risk scores for nine common and/or potentially serious adverse events related to known pharmacological properties of approximately 1200 different substances [15]. An algorithm for the summation and categorisation of each risk into no, low, intermediate or high risk, as well as clinical advice on how to handle specific risks (such as; dose reduction, discontinuation, change of medication) has been developed [15].

In this study, the following risks were included: sedation, orthostatic symptoms, constipation, anticholinergic symptoms and serotonergic symptoms, as we expected to find documentation of related symptoms in the medical records. The less common adverse events in PHARAO; QT-prolongation/cardiac arrhythmia, bleeding, seizures and renal toxicity were not included, as a low occurrence and paucity of data was expected in the present cohort.

### Extraction of clinical data

Symptoms that could possibly be associated with the pharmacological properties studied were extracted manually from the medical records data by research pharmacists. In order to ensure a conservative evaluation of serotonergic and anticholinergic symptoms, only common, and often the combination of two or more, were required for a patient to be classified as having these symptoms. A secondary reviewer checked the categorisation of included symptoms, and a senior specialist in clinical pharmacology was consulted when needed.

Finally, clinical data were linked to PHARAO risk scores (2016 version) in order to investigate how well the risk warnings from the PHARAO system correlated with documented symptoms.

### Statistical analysis

For each category of possible adverse events we calculated the sensitivity, specificity, positive predictive value, and negative predictive value of the system for high, intermediate, and low level of risk. The sensitivity refers to PHARAO’s ability to accurately identify patients who experienced symptoms possibly associated with the pharmacological properties of their medications, and was defined by the proportion of patients with true positive PHARAO alerts among those with related symptoms from their clinical data. The specificity refers to PHARAO ability to disregard any DDI or additional pharmacological effects not defined as clinically significant, and were defined by the proportion of patients with true negative PHARAO alerts among those with no related symptoms from their clinical data. The positive predictive value is the proportion of patients with PHARAO alerts who actually have the related symptoms from their clinical data. The negative predictive value is the proportion of symptoms without PHARAO alerts among those without the related symptoms from clinical data. Statistical analyses were performed using Stata version 11.1 (StataCorp, TX).

We performed a sensitivity analysis by considering only symptoms previously assessed as possible/probable/definite adverse drug reactions [20]. We also evaluated the validity of PHARAO signals when considering all prescriptions under a 6-month period, including 3 months prior to the study period used in the main analysis.

### Ethical considerations

The research was approved by the Regional Ethical Review Board in Gothenburg (no: 644–2008). Informed consent of participants was not required as the retrospective study design did not affect the healthcare of included patients. Statistics Sweden replaced the personal identity numbers by a random serial number after final data linkage and data were analysed anonymously.

## Results

The main characteristics of the study population are presented in Table 1. The mean age was 75 years (range: 65–98), and the median number of dispensed medications during the study period was 5 (range: 1–26). The study population had a median of 4 healthcare encounters and 91% of participants used primary care service only.

**Table 1:** Study population characteristics

Characteristics	
Age, median (range)	75.0 (65–98)
Female sex, *n* (%)	423 (56.8)
Number of prescribed medications, median (range)	5.0 (1–26)
1 medication, *n* (%)	81 (10.9)
2–4 medications, *n* (%)	267 (35.8)
5–9 medications, *n* (%)	290 (38.9)
≥10 medications, *n* (%)	107 (14.7)
Number of healthcare encounters, median (Q1-Q3)	4 (1–8)
Use of primary care service only, *n* (%)	680 (91.3)
Use of specialised care service^a^, *n* (%)	385 (51.7)
Hospitalisation, *n* (%)	68 (9.1)

^a^Specialised inpatient and outpatient care

Abbreviations: *Q* Quartile

In total, clinical data revealed 444 of studied symptoms in 210 patients. The most common symptoms were those possibly associated with anticholinergic drug effects, found in 180 patients, while constipation was the least common symptom, found in 25 patients (Table 2).

**Table 2:** Symptoms associated with studied PHARAO properties^a^ identified in the medical records of elderly patients

Sedation symptoms (*n* = 86)	Orthostatic symptoms (*n* = 90)	Constipation (*n* = 25)	Serotonergic symptoms (*n* = 63)	Anticholinergic symptoms (*n* = 180)
Fatigue/asthenia (66) Fall (37) Somnolence (5) Depressed level of consciousness (2)	Dizziness (59) Fall (37) Syncope (12) Orthostatic hypotension (9)	Constipation (25)	Hypertension (17) Headache (15) Tremor (12) Nausea (10) Palpitations (8) Paraesthesia (7) Diarrhoea (7) Mood altered (4) Tachycardia (4) Musculoskeletal stiffness (3) Hyperhidrosis (3) Sweating (2) Nightmare (2) Confusional state (1) Delusion (1) Libido decreased (1) Muscle spasms (1) Pyrexia (1) Restlessness (1) Sexual dysfunction (1)	Dizziness (41) Fall (23) Constipation (22) Fatigue (20) Headache (19) Anxiety (18) Decreased appetite (16) Urinary retention (13) Gastroesophageal reflux disease (13) Dementia (11) Palpitations (11) Mood altered (9) Dry mouth (8) Confusional state (7) Somnolence (4) Visual impairment (3) Memory impairment (3) Nightmare (3) Flushing (2) Pollakiuria (2) Sexual dysfunction (2) Tachycardia (2) Delusion (1) Depressed level of consciousness (1) Eye irritation (1) Hallucination (1) Irritability (1) Libido decreased (1) Vision blurred (1)

^a^In total 444 symptoms found in 210 patients

The PHARAO system generated a total of 208 high, 252 intermediate, and 316 low risk alerts, found in 127, 149 and 108 patients (out of the total study population of 745 patients), respectively (Table 3). The most common generated alerts were for constipation and anticholinergic symptoms.

**Table 3:** Validity of PHARAO alerts to detect symptoms associated with medications’ pharmacological properties during a 3-month period, by symptoms experienced by elderly patients

Experienced symptoms				Sensitivity (95% CI)	Specificity (95% CI)	PPV (95% CI)	NPV (95% CI)
According to clinical data (n)	According to PHARAO alerts						
	Lowest considered level of risk (n)^a^	PHARAO alert confirmed n (%)	Symptoms not identified by PHARAO n (%)				
Sedation (86)	High (43)	12 (28)	74 (86)	0.14 (0.08–0.23)	0.95 (0.93–0.97)	0.28 (0.16–0.44)	0.89 (0.87–0.92)
	Intermediate (60)	13 (22)	73 (85)	0.15 (0.09–0.25)	0.93 (0.90–0.95)	0.22 (0.12–0.34)	0.89 (0.87–0.91)
	Low (98)	20 (20)	66 (77)	0.23 (0.15–0.34)	0.88 (0.85–0.90)	0.20 (0.13–0.30)	0.90 (0.87–0.92)
Orthostatic symptoms (90)	High (38)	6 (16)	84 (93)	0.07 (0.03–0.15)	0.95 (0.93–0.97)	0.16 (0.07–0.32)	0.88 (0.85–0.90)
	Intermediate (120)	19 (16)	71 (79)	0.21 (0.13–031)	0.85 (0.81–0.87)	0.16 (0.10–0.24)	0.89 (0.86–0.91)
	Low (155)	24 (15)	66 (73)	0.27 (0.18–0.24)	0.80 (0.76–0.82)	0.15 (0.10–0.22)	0.89 (0.86–0.91)
Constipation (25)	High (68)	9 (13)	16 (64)	0.36 (0.19–0.57)	0.92 (0.89–0.94)	0.13 (0.07–0.24)	0.98 (0.96–0.99)
	Intermediate (136)	13 (10)	12 (48)	0.52 (0.32–0.72)	0.83 (0.80–0.85)	0.10 (0.05–0.16)	0.98 (0.96–0.99)
	Low (249)	16 (6)	9 (36)	0.64 (0.43–0.81)	0.68 (0.64–0.71)	0.06 (0.04–0.10)	0.98 (0.96–0.99)
Anticholinergic symptoms (180)	High (48)	23 (48)	157 (87)	0.13 (0.08–0.19)	0.96 (0.93–0.97)	0.48 (0.33–0.63)	0.77 (0.74–0.80)
	Intermediate (130)	44 (34)	136 (76)	0.24 (0.18–0.31)	0.85 (0.81–0.88)	0.34 (0.26–0.43)	0.78 (0.74–0.81)
	Low (246)	84 (34)	96 (53)	0.47 (0.39–0.54)	0.71 (0.67–0.75)	0.34 (0.28–0.40)	0.81 (0.77–0.84)
Serotonergic symptoms (63)	High (11)	3 (27)	60 (95)	0.05 (0.01–0.14)	0.99 (0.98–0.99)	0.27 (0.07–0.60)	0.92 (0.89–0.94)
	Intermediate (14)	4 (29)	59 (94)	0.06 (0.02–0.16)	0.98 (0.10–0.99)	0.29 (0.10–0.58)	0.92 (0.90–0.94)
	Low (28)	6 (40)	57 (90)	0.10 (0.04–0.20)	0.97 (0.95–0.99)	0.21 (0.10–0.41)	0.92 (0.90–0.94)
Total (444)^b^	High (208)	53 (25)	391 (88)	0.12 (0.09–0.15)	0.95 (0.94–0.96)	0.25 (0.20–0.32)	0.89 (0.88–0.90)
	Intermediate (460)	93 (20)	351 (79)	0.21 (0.17–0.25)	0.89 (0.88–0.90)	0.20 (0.17–0.24)	0.89 (0.88–0.90)
	Low (776)	150 (19)	294 (66)	0.37 (0.33–0.42)	0.78 (0.77–0.80)	0.20 (0.16–0.22)	0.90 (0.89–0.91)

^a^The high level of risk considers only high risk scores, intermediate level considers high and intermediate risk scores, and the low level considers high, intermediate and low risk scores. In total, 208 high, 252 intermediate, and 316 low risk alerts were found in 127, 149 and 108 patients, respectively

^b^Found in 210 patients

Abbreviations: *n* number, *NPV* negative predictive value, *PPV* positive predictive value, 95% CI = 95% confidence interval

The total specificity estimates of the PHARAO system were 0.95, 0.89 and 0.78 when we considered high, intermediate and low risk alerts, respectively. The corresponding sensitivity estimates were 0.12, 0.21 and 0.37, respectively, for each level of risk. Noteworthy, sensitivity varied markedly between the different properties with the highest value for constipation, and the lowest value for serotonergic symptoms (Table 3).

The total negative predictive value (NPV) was equal to 0.9, and the positive predictive value (PPV) was < 0.5. The highest PPV was for anticholinergic symptoms, where almost one of two high risk alerts were confirmed by clinical data.

In the sensitivity analysis (Additional file 1: Table S1 and S2), the PPV decreased significantly when only symptoms assessed as possible/probable/definite adverse drug reactions were considered. The sensitivity, specificity and the NPV did not vary or varied only slightly. The changes were in general small, and the 95% confidence intervals overlapped the main analysis.

## Discussion

Our study findings suggest that PHARAO signals have a high specificity in the studied population of relatively healthy old patients. Indeed, about eight to nine out of ten patients without clinical symptoms that could be associated with the studied pharmacological properties did not generate any signal from the system. Further, about nine out of ten patients without PHARAO signals were confirmed not to have such symptoms from their clinical data, according to the negative predictive value of our analysis.

The sensitivity, on the other hand, was in general rather low, and varied between the different pharmacological properties. Overall sensitivity values between 0.12 and 0.37 (from high to low risk level) indicate that about 80% of patients who actually experienced symptoms possibly associated with PHARAO properties did not generate a signal. This is to be expected, as there may be many other causes for patients’ symptoms than drug treatment, but it warrants more research to ensure that most individuals with symptoms caused by drugs are captured.

Constipation signals had the highest sensitivity, while signals for possible serotonergic symptoms had the lowest. In this context, constipation is a symptom that is easily interpreted and classified from patients´ medical records. On the other hand, symptoms possibly related to serotonergic over activity are much more unspecific and difficult to classify, and misdiagnosis is likely to be high [21, 22], even though we aimed to have a conservative categorisation of clinical symptoms belonging to this group. Moreover, it is unclear to what extent additive serotonergic effects from several drugs will cause a gradual increase in the frequency of adverse events. Notwithstanding, anticholinergic symptoms, although often unspecific, had a much higher sensitivity level, with nearly half of the symptoms confirmed by a PHARAO signal. This may relate to a more direct relation between cholinergic antagonism and experienced adverse events by patients [23].

The low positive predictive values of the PHARAO signals may probably be related to the low prevalence of the studied adverse drug events in this relatively healthy cohort of old adults, as compared to studies conducted among hospitalized patients.

### Strengths and limitations

The main strength of this research is that it provides a scientific knowledge concerning the clinical relevance of pharmacological risk assessments integrated into a CDSS, based on older patients’ experienced symptoms, as documented in their medical records. As far as we know, such information is scarce in the scientific literature [10]. We aimed in our study to use real-life clinical data, as naturalistic studies are considered to have an important role in evaluating the validity of CDSSs [10]. Furthermore, PHARAO scores are based on the whole list of the prescribed drugs for each patient, which reflects the situation that may face the prescriber in practice, when more than two medications may possibly interact, and which are often not considered in other alert systems. Another strength of the present study is the consideration of less serious symptoms, such as dizziness, fatigue and constipation, that are rarely investigated, but that may still affect the quality of life of many patients, and the benefit-harm assessment of the treatment [24].

The Swedish Prescribed Drug Register has a full coverage of prescriptions dispensed in pharmacies and nursing homes, and is therefore a reliable source to study drug exposure at the population level. Nonetheless, this register does not include medications prescribed during hospital care [18]. However, only one out of ten patients was subject to hospital care during the study period, and we do not expect that medicines administered only during hospital stay would have a significant impact on the results of this study. Moreover, register data may overestimate the risk of drug interactions, as patients may not have taken all their prescribed medications concomitantly, or prescribers may have adjusted the doses to lower the risk of DDIs. Furthermore, we studied PHARAO alerts of medications prescribed only during the study period while patients may have experienced symptoms associated with prescribed medication refilled prior to the study period. However, when we performed a sensitivity analysis during a six-month period, including three months prior the study, the results varied only marginally. Noteworthy, patients may experience serious adverse drug events caused by only one medication. However, no medium or high alerts would be generated by PHARAO, as higher scores require the combination of at least two medications. Only one out of ten patients were dispensed a single medication during the study period, so this should only marginally affect our study findings.

The validity of PHARAO alerts was evaluated based on patients’ symptoms extracted from their medical records. The dataset was generated in 2008, meaning that medicines introduced later are not included, which is a limitation. The PHARAO version used was from 2016. New drugs are regularly added to the system, but older ones are not removed, meaning that all drugs present in the clinical material were also present in the PHARAO system of 2016. Many new drugs introduced in the period in between have been for use in specialist care, such as oncology. We find it unlikely that the presence of these new medicines will significantly change the performance of PHARAO in an outpatient setting.

The quality of medical records data may also have influenced our results. Symptoms not communicated by patients or not recorded by caregivers could not be identified in this study, as well as symptoms not recognised as relevant during the independent data extraction. Moreover, symptoms included in our study may not be associated with the pharmacological properties of prescribed medications, but rather be symptoms of a medical condition. We purposefully included all experienced symptoms during the study period, without a preliminary causality assessment to reflect the clinical practice, as practitioners often fail to recognise mild or minor adverse drug events, in particular among the old adults with multiple chronic conditions [25]. We had similar results when we only included patients previously assessed to have possible, probable, or definite adverse drug reactions.

The categorisation of anticholinergic and serotonergic symptoms was somehow challenging, since, to our knowledge, there is no standard rule for this. For example, more structured decision rules to diagnose serotonergic symptoms have been suggested. However, they are mainly applied to severe cases, and their applicability to milder symptoms in clinical practice is unknown [26].

### Comparison to previous research

To the best of our knowledge, this study is the first to evaluate how well the risk signals from a CDSS correlate with real experienced symptoms identified from clinical data. Previous studies that evaluated the predictive values of DDI alerts, either compared the alerts generated by the system with pairs of interacting medications, chosen by consensus among the researchers [27–30], or were based on expert panel’s estimates of the probability that the DDI would result in an adverse drug event [31]. These studies have found mixed results on the values of sensitivity and specificity. However, direct comparison between different interaction warning systems is difficult since the interactions included and the classification of interaction severity differs across different CDSSs [10, 32].

#### Implications

An identified high specificity of the PHARAO system supports its usefulness as a screening tool, in combination with a regular DDI database, for possible drug synergies as the cause of adverse events in patients, while maintaining a low risk of false positive alarms. In clinical practice, the use of such system is often preferred by prescribers to avoid the risk of alert-fatigue, leading to the systematic override of warnings [12, 33, 34]. The low positive predictive value for the signals indicates that an additional evaluation of the cause of symptoms has to be performed. Ancillary measures should always be used in conjunction, such as the integration of individual patient comorbidities and medical history, in order to consider other possible alternate causes of experienced symptoms [35].

This study examined the validity of PHARAO alerts to detect actual adverse symptoms experienced by the old patients. Future studies need to determine the acceptable sensitivity and specificity values in clinical practice and to investigate prescribers’ views on PHARAO usefulness during the prescribing and the medication review process (10).

## Conclusions

In conclusion, PHARAO system has a high specificity and a low sensitivity to detect symptoms potentially associated with pharmacological properties of patients’ prescribed medications. The use of such system may decrease the alert fatigue, but other clinical data, such as drug doses, should be considered to improve the sensitivity of the system in clinical practice.

## Additional file

Additional file 1: Sensitivity analysis. **Table S1.** Validity of PHARAO alerts to detect symptoms associated with medications’ pharmacological properties, related to symptoms assessed as possible/probable/definite adverse drug reactions. **Table S2.** Validity of PHARAO alerts to detect symptoms associated with medications’ pharmacological properties during a 6-month period, by symptoms experienced by elderly patients. (DOCX 16 kb)

## Data Availability

The datasets used and analysed during the current study are available from the corresponding author on reasonable request.

## Abbreviations

CDSS: Computerised decision support system
DDIs: Drug-drug interaactions
NPV: Negative predictive value
PPV: Positive predictive value

## References

[1] Klarin I, Wimo A, Fastbom J (2005). The association of inappropriate drug use with hospitalisation and mortality. Drugs Aging.

[2] Pirmohamed M, James S, Meakin S, Green C, Scott AK, Walley TJ (2004). Adverse drug reactions as cause of admission to hospital: prospective analysis of 18 820 patients. Bmj..

[3] Corsonello A, Pedone C, Incalzi RA (2010). Age-related pharmacokinetic and pharmacodynamic changes and related risk of adverse drug reactions. Curr Med Chem.

[4] Maher RL, Hanlon J, Hajjar ER (2014). Clinical consequences of polypharmacy in elderly. Expert Opin Drug Saf.

[5] Hovstadius B, Hovstadius K, Åstrand B, Petersson G (2010). Increasing polypharmacy-an individual-based study of the Swedish population 2005-2008. BMC Clin Pharmacol.

[6] Juurlink DN, Mamdani M, Kopp A, Laupacis A, Redelmeier DA (2003). Drug-drug interactions among elderly patients hospitalized for drug toxicity. Jama..

[7] Rosas-Carrasco Ó, García-Peña C, Sánchez-García S, Vargas-Alarcón G, Gutiérrez-Robledo LM, Juárez-Cedillo T (2011). The relationship between potential drug-drug interactions and mortality rate of elderly hospitalized patients. Rev Investig Clin.

[8] Roshanov PS, Fernandes N, Wilczynski JM, Hemens BJ, You JJ, Handler SM (2013). Features of effective computerised clinical decision support systems: meta-regression of 162 randomised trials. Bmj..

[9] Robertson J, Walkom E, Pearson SA, Hains I, Williamson M, Newby D (2010). The impact of pharmacy computerised clinical decision support on prescribing, clinical and patient outcomes: a systematic review of the literature. Int J Pharm Pract.

[10] Coleman JJ, van der Sijs H, Haefeli WE, Slight SP, McDowell SE, Seidling HM (2013). On the alert: future priorities for alerts in clinical decision support for computerized physician order entry identified from a European workshop. BMC medical informatics and decision making.

[11] Metzger J, Welebob E, Bates DW, Lipsitz S, Classen DC (2010). Mixed results in the safety performance of computerized physician order entry. Health Aff.

[12] Van Der Sijs H, Aarts J, Vulto A, Berg M (2006). Overriding of drug safety alerts in computerized physician order entry. J Am Med Inform Assoc.

[13] Moxey A, Robertson J, Newby D, Hains I, Williamson M, Pearson S-A (2010). Computerized clinical decision support for prescribing: provision does not guarantee uptake. J Am Med Inform Assoc.

[14] Böttiger Y, Laine K, Andersson ML, Korhonen T, Molin B, Ovesjö M-L (2009). SFINX—a drug-drug interaction database designed for clinical decision support systems. Eur J Clin Pharmacol.

[15] Böttiger Y, Laine K, Korhonen T, Lähdesmäki J, Shemeikka T, Julander M (2018). Development and pilot testing of PHARAO—a decision support system for pharmacological risk assessment in the elderly. Eur J Clin Pharmacol.

[16] Hakkarainen KM, Gyllensten H, Jönsson AK, Andersson Sundell K, Petzold M, Hägg S (2014). Prevalence, nature and potential preventability of adverse drug events–a population-based medical record study of 4970 adults. Br J Clin Pharmacol.

[17] Ludvigsson JF, Otterblad-Olausson P, Pettersson BU, Ekbom A (2009). The Swedish personal identity number: possibilities and pitfalls in healthcare and medical research. Eur J Epidemiol.

[18] Wettermark B, Hammar N, MichaelFored C, Leimanis A, Otterblad Olausson P, Bergman U (2007). The new Swedish prescribed drug register—opportunities for pharmacoepidemiological research and experience from the first six months. Pharmacoepidemiol Drug Saf.

[19] Wiréhn A-BE, Karlsson HM, Carstensen JM (2007). Estimating disease prevalence using a population-based administrative healthcare database. Scandinavian J Public Health.

[20] Hedna K, Hakkarainen KM, Gyllensten H, Jönsson AK, Petzold M, Hägg S (2015). Potentially inappropriate prescribing and adverse drug reactions in the elderly: a population-based study. Eur J Clin Pharmacol.

[21] Mackay F, Dunn N, Mann R (1999). Antidepressants and the serotonin syndrome in general practice. Br J Gen Pract.

[22] Frank C (2008). Recognition and treatment of serotonin syndrome. Can Fam Physician.

[23] Mintzer J, Burns A (2000). Anticholinergic side-effects of drugs in elderly people. J R Soc Med.

[24] Gandhi TK, Burstin HR, Cook EF, Puopolo AL, Haas JS, Brennan TA (2000). Drug complications in outpatients. J Gen Intern Med.

[25] Rochon PA, Gurwitz JH (1997). Optimising drug treatment for elderly people: the prescribing cascade. Bmj..

[26] Dunkley E, Isbister G, Sibbritt D, Dawson A, Whyte I (2003). The hunter serotonin toxicity criteria: simple and accurate diagnostic decision rules for serotonin toxicity. Qjm..

[27] Saverno KR, Hines LE, Warholak TL, Grizzle AJ, Babits L, Clark C (2010). Ability of pharmacy clinical decision-support software to alert users about clinically important drug—drug interactions. J Am Med Inform Assoc.

[28] Abarca J, Colon LR, Wang VS, Malone DC, Murphy JE, Armstrong EP (2006). Evaluation of the performance of drug-drug interaction screening software in community and hospital pharmacies. J Manag Care Pharm.

[29] Ing KL, Reutemann B, Samer C, Guignard B, Bonnabry P, Dayer P (2012). Comparison of four drug interaction screening programs. Revue medicale suisse.

[30] Hazlet TK, Lee TA, Hansten PD, Horn JR (2001). Performance of community pharmacy drug interaction software. J Am Pharm Assoc (1996).

[31] Weingart SN, Simchowitz B, Padolsky H, Isaac T, Seger AC, Massagli M (2009). An empirical model to estimate the potential impact of medication safety alerts on patient safety, health care utilization, and cost in ambulatory care. Arch Intern Med.

[32] Wolfstadt JI, Gurwitz JH, Field TS, Lee M, Kalkar S, Wu W (2008). The effect of computerized physician order entry with clinical decision support on the rates of adverse drug events: a systematic review. J Gen Intern Med.

[33] van der Sijs H, Mulder A, van Gelder T, Aarts J, Berg M, Vulto A (2009). Drug safety alert generation and overriding in a large Dutch university medical Centre. Pharmacoepidemiol Drug Saf.

[34] Isaac T, Weissman JS, Davis RB, Massagli M, Cyrulik A, Sands DZ (2009). Overrides of medication alerts in ambulatory care. Arch Intern Med.

[35] Troiano D, Jones MA, Smith AH, Chan RC, Laegeler AP, Le T (2013). The need for collaborative engagement in creating clinical decision-support alerts. Am J Health Syst Pharm.

