# Supplementary material for: Clinical relevance of alerts from a decision support system, PHARAO, for drug safety assessment in the older adults
Source: BMC Geriatr. 2019 Jun 11;19:164. doi: 10.1186/s12877-019-1179-y (PMC6560851; doi:10.1186/s12877-019-1179-y)
Supplement: Supplementary file 1 — Sensitivity analysis. Table S1. Validity of PHARAO alerts to detect symptoms associated with medications’ pharmacological properties, related to symptoms assessed as possible/probable/definite adverse drug reactions. Table S2. Validity of PHARAO alerts to detect symptoms associated with medications’ pharmacological properties during a 6-month period, by symptoms experienced by elderly patients. (DOCX 16 kb) [file 12877_2019_1179_MOESM1_ESM.docx]

**Clinical relevance of alerts from a decision support system, PHARAO, for drug safety assessment in the old adults**

**Additional file 1. Sensitivity analysis**

**Table S1. Validity of PHARAO alerts to detect symptoms associated with medications’ pharmacological properties, related to symptoms assessed as possible/probable/definite adverse drug reactions**

| Adverse drug reactions | | | | Sensitivity  (95% CI) | Specificity  (95% CI) | PPV  (95% CI) | NPV  (95% CI) |
| --- | --- | --- | --- | --- | --- | --- | --- |
| According to clinical data* (n) | According to PHARAO alerts | | |  |  |  |  |
|  | Lowest considered level of alert (n)** | PHARAO alert confirmed n (%) | Symptoms not identified by PHARAO n (%) |  |  |  |  |
| **Total (208)** | High (208) | 22 (11) | 186 (89) | 0.11 (0.07-0.15) | 0.95 (0.94-0.95) | 0.11 (0.07-0.16) | 0.95 (0.94-0.96) |
|  | Intermediate (460) | 43 (9) | 165 (79) | 0.21 (0.16-0.27) | 0.88 (0.87-0.89) | 0.09 (0.07-0.12) | 0.95 (0.94-0.96) |
|  | Low (907) | 68 (7) | 140 (67) | 0.33 (0.26-0.40) | 0.77 (0.76-0.78) | 0.07 (0.06-0.09) | 0.95 (0.94-0.96) |

*Symptoms considered as possible/probable/definite adverse drug reactions, following a causality assessment were included in the analysis.

** The high level of risk considers only high risk scores, intermediate level considers high and intermediate risk scores, and the low level considers high, intermediate and low risk scores.

Abbreviations: n = number; NPV = negative predictive value; PPV = positive predictive value; 95%CI = 95% confidence interval

**Table S2. Validity of PHARAO alerts to detect symptoms associated with medications’ pharmacological properties during a 6-month period, by symptoms experienced by elderly patients.**

| Experienced symptoms | | | | Sensitivity  (95% CI) | Specificity  (95% CI) | PPV  (95% CI) | NPV  (95% CI) |
| --- | --- | --- | --- | --- | --- | --- | --- |
| According to clinical data (n) | According to PHARAO alerts* | | |  |  |  |  |
|  | Lowest level of alert considered (n)** | PHARAO alert confirmed n (%) | Symptoms not identified by PHARAO n (%) |  |  |  |  |
| **Total (444)** | High (313) | 72 (23) | 372 (84) | 0.16 (0.13-0.20) | 0.89 (0.88-0.90) | 0.23 (0.18-0.28) | 0.92 (0.91-0.93) |
|  | Intermediate (602) | 119 (20) | 325 (73) | 0.27 (0.23-0.31) | 0.85 (0.84-0.86) | 0.20 (0.17-0.23) | 0.89 (0.90-0.91) |
|  | Low (945) | 169 (18) | 275 (62) | 0.38 (0.33-0.43) | 0.76 (0.75-0.78) | 0.18 (0.15-0.20) | 0.90 (0.89-0.91) |

*PHARAO risk scores calculated under a six-month period, including 3 months prior the study period.

** The high level of risk considers only high risk scores, intermediate level considers high and intermediate risk scores, and the low level considers high, intermediate and low risk scores.

Abbreviations: n = number; NPV = negative predictive value; PPV = positive predictive value; 95%CI = 95% confidence interval
